# Association of frailty with workplace social activity, physical activity, and well-being among older employees: a moderated mediation in two income-variant samples

**DOI:** 10.1186/s12877-024-05178-9

**Published:** 2024-07-03

**Authors:** Emelia Danquah, Nestor Asiamah, Reginald Arthur-Mensah Jnr, Kyriakos Kouveliotis

**Affiliations:** 1 Research Directorate, Koforidua Technical University, E/R, Koforidua, Ghana; 2 Division of Interdisciplinary Research and Practice, School of Health and Social Care, University of Essex, Colchester, Essex, CO4 3SQ UK; 3 Department of Gerontology and Geriatrics, Africa Centre for Epidemiology, P.O. Box AN 18462, Accra North, Accra, Ghana; 4 Department of Nursing and Midwifery, Faculty of Health and Allied Sciences, Pentecost University, P.O. Box KN 1739, Accra, Ghana; 5 Berlin School of Business and Innovation, Academic Affairs, 97-99 Karl Marx Strasse, 12043 Berlin, Germany

**Keywords:** Frailty, Physical activity, Income, Workplace social activity, Older adults, Well-being

## Abstract

**Background:**

Research suggests that frailty is associated with lower physical activity and well-being in old age, but social activities at work may facilitate physical activity and its positive effect on well-being among older employees with frailty. This study, therefore, ascertained whether there is a moderated mediation of the association of frailty, Workplace Social Activity (WSA), and well-being by Physical Activity (PA).

**Methods:**

The study adopted a cross-sectional design with relevant sensitivity analyses for confounding. The participants were within two Ghanaian samples with different income levels (low-income, *n* = 897, and higher income, *n* = 530). The minimum samples were calculated, and the statistical models were tested with Haye’s Process Model through structural equation modelling.

**Results:**

Frailty was negatively associated with PA, and this relationship was moderated by WSA in both samples. Higher frailty was directly and indirectly associated with lower well-being in the higher-income sample but only indirectly associated with lower well-being in the low-income sample. The mediation of PA in the frailty-well-being relationship is partial in the higher-income sample but complete in the low-income sample. There was evidence of moderated mediation in both samples.

**Conclusion:**

WSA may reduce the strength of the negative association of frailty with PA and well-being among older employees in both samples. Workplace interventions aimed at enhancing WSA may encourage PA and enhance well-being among older employees with frailty.

**Supplementary Information:**

The online version contains supplementary material available at 10.1186/s12877-024-05178-9.

## Introduction

Frailty is physical impairments and limitations of daily physical activities, including Activities of Daily Living (ADLs) and Instrumental Activities of Daily Living (IADLs) [1, 2]. Though several definitions exist, we adopt this definition because it agrees with our measurement of frailty in a clinical context to proffer implications for health service delivery. More so, ADLs and IADLs are primary tasks by which older adults’ daily functioning and Physical Activity (PA) are ideally assessed. As such, the above definition provides a suitable context for any study examining the link between frailty and PA among older adults.

Research has shown that several factors influence frailty, but the most dominant factor is ageing [3, 4]. As population ageing intensifies, we can expect the burden of frailty and its healthcare needs to increase. Against this backdrop, interventions enabling ageing people to delay the onset or progression of frailty are needed. Any programme enabling individuals to maintain PA over the life course could be one of these interventions since previous research [5–7] has confirmed the effectiveness of PA in delaying or lowering frailty. On the flip side, frailty is often associated with functional difficulties that prevent people from performing PA [6, 8], given that people with frailty may not have enough physical strength to perform PA. This potential negative effect of frailty on PA is one of the foci of this study.

The literature suggests that frailty and its associated functional limitations can create feelings of unwellness [9–11], which is analogous to the direct negative effect of frailty on well-being. Research has also established that PA is associated with well-being [7, 12, 13], which means that higher PA is associated with higher well-being. Thus, the negative effect of frailty on well-being and PA, and the positive effect of PA on well-being imply a potential mediation of the relationship between frailty and well-being by PA. An assessment of this mediation role is necessary because the effect of PA on well-being may not be as positive as the literature suggests in a situation where it is negatively influenced by frailty. Similarly, frailty may have an indirect positive effect on well-being through PA, given the positive effect of PA on well-being.

Employees, especially those with full-time jobs, spend an average of 8 h at work [14, 15], which means that full-time employees spend most of their waking time at work during a weekday. As such, employment provides an opportunity to engage in sufficient Workplace Social Activity (WSA), which we define as participation in social activities with peers, the provision of social support to others, and volunteering to advance organizational interests. This definition draws on a previous study [16], which operationalised social activity as participation in group activities, providing social support to others, and volunteering in the community. We adopt this definition because its three domains are recognised as activities performed frequently at work that yield desirable outcomes for organizations and their employees [17–19].

Social activity provides opportunities for performing PA [4, 20–22], and some social activities such as group walks and playing games with others constitute PA. If so, social activity can buffer frailty while increasing PA. This reasoning is corroborated by studies [22–24] showing that social activity is associated with lower frailty. We infer based on this thought that WSA can modify the effect of frailty on PA and, as a result, modify the above-mentioned mediation of PA in the frailty-well-being relationship. Yet, no study has examined this relationship analogous to a moderated mediation by PA in the nexus between frailty, WSA, and well-being. This study aimed to test this potential moderated mediation by answering the following research questions: (1) does frailty have a direct effect on well-being, (2) does PA mediate the effect of frailty on well-being, and (3) is there a moderated mediation by PA in the relationship between frailty, WSA, and well-being?

This study is novel in several ways. As mentioned earlier, there is no evidence regarding how PA affects well-being when it is limited by frailty. This study is the first to attempt to provide this evidence by testing the potential mediation of PA in the frailty-well-being relationship. It is also the first to test the above moderated mediation. An examination of this nexus in a work context is significant because the proportion of older employees is expected to increase as population ageing intensifies, and more empirical evidence is needed to develop workplace policies for promoting PA and WSA in organizations in the interest of employees’ healthy ageing. Income is one of the strongest predictors of frailty [3, 25], which means that the above moderated mediation can differ between employee groups with different income levels. This study, therefore, assessed the moderated mediation between low- and higher-income employee samples to advance the evidence to date and provide implications for workplace policies.

**Fig. 1:**
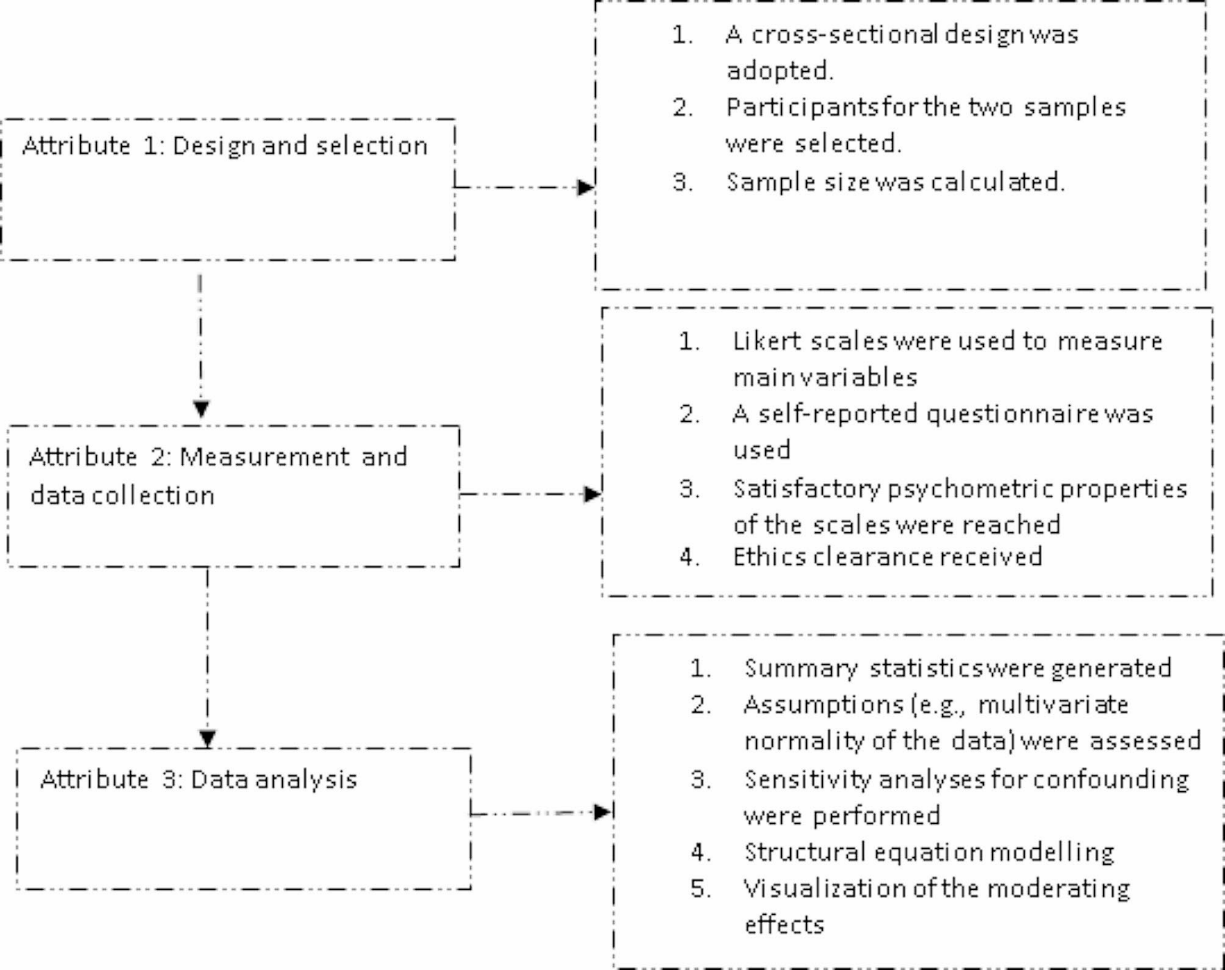
Attributes of the study design

## Methods

### Design

A cross-sectional design was adopted. This design comprises a sensitivity analysis against confounding and other measures compliant with the STROBE (i.e., Strengthening The Reporting of Observational Studies in Epidemiology). Figure 1 is a flowchart of the study design.

### Participants and their selection

The participants of this study were individuals working in Accra, Ghana. To meet the aim of this study, the following three inclusion criteria were utilised to select the participants: (i) having a minimum of a basic education qualification, which we used to assess the ability to complete the questionnaire in English, (ii) being an employee aged 50 years or older, and (iii) availability and willingness to participate in the study. We obtained two samples with different income levels by selecting the participants from two communities in Accra. The low-income sample came from low socioeconomic areas where the population density was high, roads were either untarred or unrepaired, and most residents were low-income earners (e.g., petty traders, and low or middle-level employees). The higher-income sample was from high socioeconomic areas where the streets were interconnected and well-tarred, and most of the residents were high-income earners (e.g., business executives, senior managers, and professors).

A total of 967 and 677 eligible individuals met the inclusion criteria and were selected from the low and high socio-economic areas respectively. Individuals were selected through structured interviews at supermarkets and community centres. The minimum sample required for our analysis was calculated with relevant statistics and parameters (i.e., [α = 0.05, power = 0.8, effect size = 0.2, and a maximum of 10 independent variables) from previous research [26, 27]. The sample size reached through the G*Power software was 91. Data were collected on all the eligible participants to maximise the statistical power of the tests. With an independent samples t-test, we confirmed that the higher-income sample (mean = 2515.48; standard deviation = 107.31) had a larger average income, compared to the low-income sample (mean = 1118.19; standard deviation = 23.12) at a statistical significance of *p* < 0.001.

### Variables and measurement

WSA was measured with an 8-item scale adopted in whole from a previous study [28] with its four descriptive anchors (not at all – 1, sometimes – 2, often – 3, always – 4). This tool measures how often the individual engaged in three domains of social activity (i.e., social or peer support, participating in group activities, and volunteering) at work. This scale was used because it is relatively brief, is the only available measure of WSA, and focuses on older adults. It produced a satisfactory internal consistency in the form of Cronbach’s α ≥ 0.7 (low-income sample, α = 0.86 and higher-income sample, α = 0.87). Scores of the WSA scale range from 8 to 32, with larger scores indicating higher workplace social activity.

Frailty was measured with the 15-item Tilburg Frailty Index that was borrowed in whole from the literature [1]. It accompanies two descriptive anchors (i.e., no – 0, and yes – 1) and measures frailty in a clinical context. We preferred this tool to other related tools because it measures frailty in a clinical context and, thus, enabled us to meet our goal of identifying possible implications for health service delivery. It produced satisfactory Cronbach’s α ≥ 0.7 on both samples (i.e., low-income, α = 0.82, and higher-income, α = 0.89). The scale’s scores range from 0 (no frailty) to 15 (highest frailty).

Well-being was measured with the World Health Organization’s 5-item well-being scale, which has five descriptive anchors (never – 1, sometimes – 2, often – 3, very often – 4, and all the time – 5). This measure was adopted in whole from a previous study [29] and was internally consistent at a minimum of Cronbach’s α ≥ 0.7 (low-income, α = 0.93, and higher-income, α = 0.95). The scores of the scale range from 5 to 25, with higher scores indicating higher well-being.

PA was measured with the exercise behaviour domain of the Health-promoting Behaviour Scale, which was adopted in whole with its four descriptive anchors (i.e., 1 – never, 2 – sometimes, 3 – often, and 4 – routinely) from a recent study [30]. This tool measures how often the individual performed moderate and vigorous PA over the past 30 days. We preferred it to other measures of PA because it is brief, free of cost, and less susceptible to recall bias. Other subjective scales (e.g., International Physical Activity Questionnaire) are more vulnerable to recall bias among older participants since they ask for specific times spent exercising over a period. Moreover, we did not have enough funds to use objective measures such as activity trackers. The scale produced satisfactory internal consistency in the form of Cronbach’s α ≥ 0.7 (low-income, α = 0.92, and higher-income, α = 0.95). Scores of the PA scale range from 5 to 20, with higher scores indicating higher PA. Following previous research, data on the above variables were formed by summing their items. Appendix 1 shows items of the scales used.

We further measured 7 personal variables as potential covariates. Three of the variables (i.e., age, education, and income) were measured as discrete variables. Education was measured by asking the participants to report their years of schooling. Income was the participant’s gross monthly income in Ghana cedis, and age was a measure of chronological age. Sex (males – 1, and females – 2), marital status (not married – 1, and married – 2), self-reported health (poor – 1, and good – 2), and chronic disease status were measured as dichotomous categorical variables. Chronic disease status was measured by asking participants to report chronic disease conditions they had. Responses were coded into two groups (none – 1, and one or more – 2). All categorical variables were codded into dummy-type variables for our statistical analysis.

### Research instrument

We utilised a self-reported questionnaire to gather data. The questionnaire had a preamble including the survey completing instructions. Scales or measures on the main variables (i.e., frailty, PA, WSA, and well-being) were presented first, followed by questions on the covariates and demographic variables. Unique information and instructions in distinct subsections were provided for completing each scale and the demographic variables. Finally, we followed the standard procedures recommended in the literature [27, 31] to assess Common Methods Bias (CMB). These procedures included Herman’s one-factor procedure [31] in the form of an Exploratory Factor Analysis (EFA) with varimax rotation based on each scale. In the EFA, the variance of the first factor or the entire scale (i.e., for scales with a unidimensional factor structure) was less than 40% as recommended [31]. This result confirmed the absence of CMB in the data. Appendix 2 shows variances explained in EFA for each scale.

### Data collection

Ethics review and clearance were received from the ethics committee of the Africa Centre for Epidemiology in Accra (review number: 001-2023-ACE). Written informed consent was provided by all the participants, and we followed all relevant ethical procedures based on the Declaration of Helsinki. Questionnaires were administered at locations where the participants were recruited with the support of four research assistants. Data collection was coordinated by two of the researchers in Accra. In situations where the participants could not respond instantly, they were given up to two weeks to return completed questionnaires through couriers hired by the researchers. Data were gathered over four weeks (i.e., April 1–30, 2023). After discarding questionnaires that were filled halfway, we analysed 897 questionnaires for the low-income sample and 530 questionnaires for the higher-income sample.

**Fig. 2:**
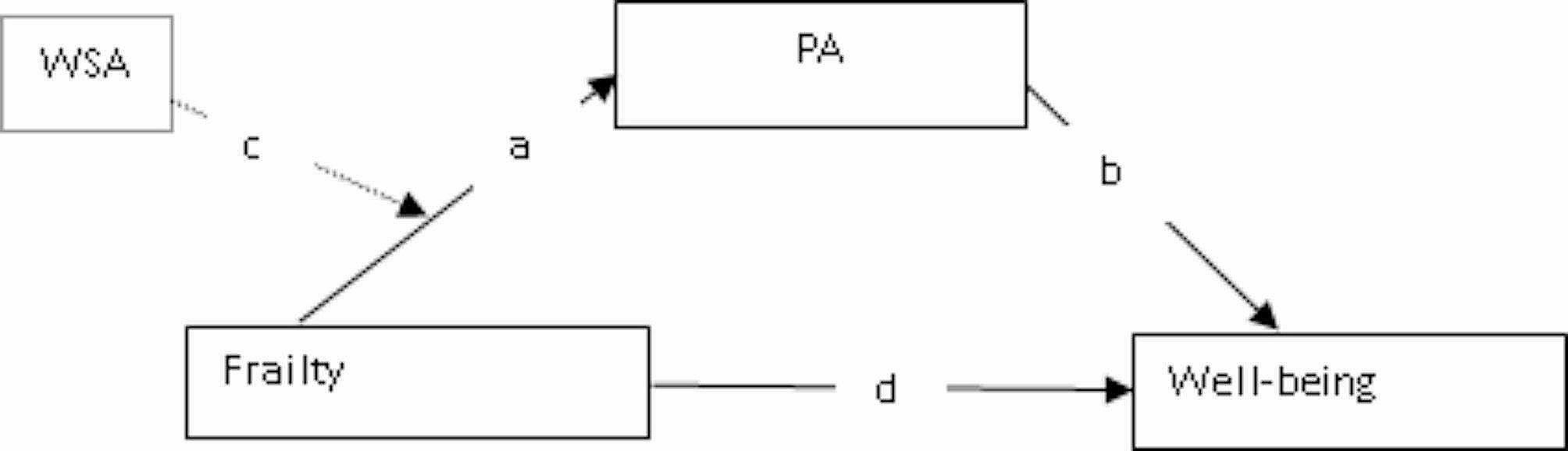
A conceptual framework of the moderated mediation by physical activity. *Note* WSA – workplace social activity; PA – physical activity; a – effect of frailty on physical activity; b – effect of physical activity on wellbeing; c – workplace social activity moderates the effect of frailty on physical activity, d –effect of frailty on wellbeing

### Statistical analysis technique

Data were analysed to test the conceptual model shown in Fig. 2. SPSS 29 (IBM Inc. New York, USA) and Amos 29 were utilised to analyse the data. SPSS was used in the first phase of the analysis to perform exploratory data analysis whereas Amos was used in the second phase to test the moderated mediation model through Structural Equation Modelling (SEM). In the first phase, descriptive statistics were generated on all variables to summarise the data and identify the proportion of missing data associated with each variable. Only three variables (i.e., gender, marital status, and self-reported health) had missing data, and the missing data were less than 10%. As such, we analysed the data with the missing data based on previous research [27, 32]. Another aspect of the exploratory analysis was our sensitivity analysis for the ultimate confounding variables. This procedure was adopted from previous studies [27, 32] and was aimed at screening the measured potential covariates for the ultimate confounders that would significantly confound the primary relationships tested. With this procedure, we avoided incorporating irrelevant variables into the moderated mediation model as covariates. None of the measured potential covariates qualified as an ultimate confounder, and Appendix 3a shows steps taken in this analysis.

**Fig. 3:**
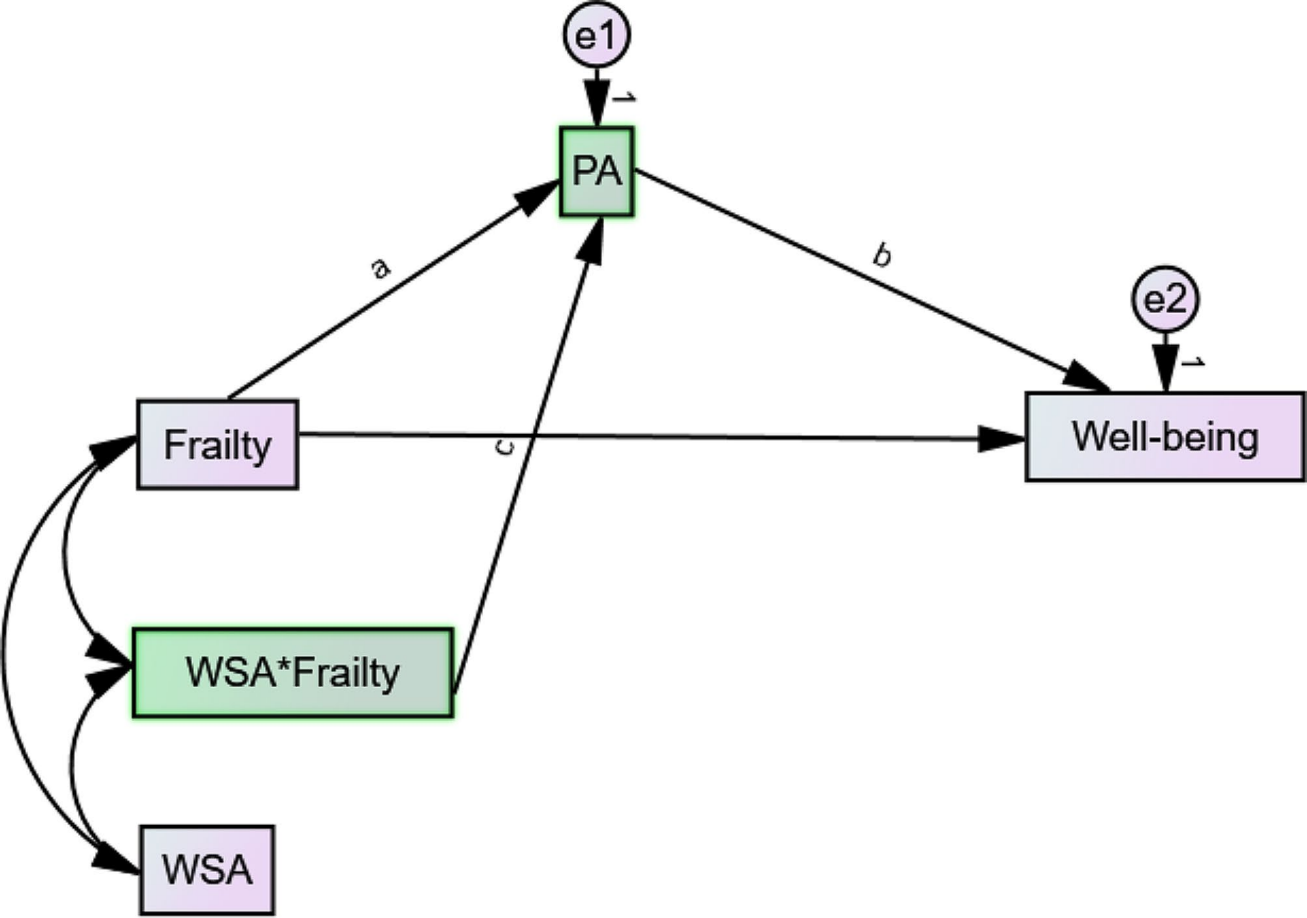
A statistical model of the moderated mediation by physical activity. *Note* PA – physical activity; WSA – workplace social activity

We analysed data in phase 2 to address the research questions by fitting a moderated mediation model based on Haye’s Process [33]. A part of this model was a variable representing the interaction between WSA and frailty (i.e., WSA*frailty). To create this interaction term, we mean-centred the moderating variable (i.e., WSA) and used the compute function to multiply it by frailty. Figure 3 shows the statistical moderated-mediated model fitted with the low- and higher-income samples. In fitting this model according to Haye’s Process, we utilised the “user-defined estimands” function to estimate the Simple Slope (SS), Conditional Indirect Effect (CIE), and Index of Moderated Mediation (InModMed). The SS and CIE were estimated at different levels (i.e., low, middle, high) of the moderating variable, WSA. Appendix 3b shows the equations used to compute these indexes. The model was fitted based on 2000 bias-corrected bootstraps at a 95% confidence level. The bootstraps compensated for our data’s violation of the assumption of normal distribution [34]. The moderating effects for the low- and higher-income samples were depicted in Figs. 2 and 3 respectively. The statistical significance of the results was detected at *p* < 0.05.

**Table 1:** Descriptive and summary statistics on variables

Variable	Group	Low income		Higher income	
		*n*/mean	%/SD	*n*/mean	%/SD
Categorical variables					
Gender	Men	465	51.84	271	51.13
	Women	411	45.82	259	48.87
	Missing	21	2.34	---	---
	Total	897	100.00	530	100.00
Chronic disease status	None	123	13.71	316	59.62
	≥ 1	774	86.29	214	40.38
	Total	897	100.00	530	100.00
Marital status	Not married	240	26.76	104	19.62
	Married	576	64.21	414	78.11
	Missing	81	9.03	12	2.26
	Total	897	100.00	530	100.00
Self-reported health	Poor	291	32.44	204	38.49
	Good	552	61.54	322	60.75
	Missing	54	6.02	4	0.75
	Total	897	100.00	530	100.00
Discrete variables					
Frailty	---	5.57	3.14	6.69	2.74
Physical activity	---	10.21	2.53	10.96	3.19
Well-being	---	13.29	3.93	14.69	4.82
Workplace Social Activity	---	17.56	4.20	19.20	6.15
Income (C)	---	1118.19	23.12	2515.48	107.31
Age (yrs)	---	61.56	41.38	59.32	8.13
Education (yrs)	---	17.02	4.65	16.92	3.29

*Note* SD – standard deviation (of the mean); n – frequency; mean and SD apply to discrete variables whereas frequency and % apply to categorical variables; --- not applicable

## Results

In Table 1, about 52% (*n* = 465) of the participants were men in the low-income sample whereas 51% (*n* = 271) were men in the higher-income sample. The averages of income in the low- and higher-income samples were about 1118 (Mean = 1118.19; SD = 23.12) and 2515 (Mean = 2515.48; SD = 107.31) respectively. The mean age of the low-income sample was about 62 years (Mean = 61.56; SD = 41.38) whereas that of the higher-income sample was about 59 years (Mean = 59.32; SD = 3.29). The average years of schooling were about 17 years in both samples. In Ghana, pre-tertiary education (which takes close to 17 years to complete) is affordable for all income groups, which explains why the two samples had similar average years of schooling.

The averages of frailty in the low and higher-income samples were about 6 (Mean = 5.57; SD = 3.14) and 7 (Mean = 6.69; SD = 2.74) respectively.

**Table 2:** Associations of frailty and physical activity with well-being in the two samples

Outcome	Path	Direct effects							Indirect effects	
		Predictor	B	β	SE (of B)	Critical ratio	*p*	Label	β	*p*
Low-income (*n* = 897)										
PA	<---	Frailty	-0.128	-0.140	0.025	-5.014	***	a		
Well-being	<---	PA	0.683	0.477	0.042	16.347	***	b		
Well-being	<---	Frailty	-0.021	-0.016	0.038	-0.552	0.581		-0.067	***
PA	<---	WSA*frailty	0.043	0.489	0.002	17.473	***	c		
Higher income (*n* = 530)										
PA	<---	Frailty	-0.187	-0.160	0.046	-4.020	***	a		
Well-being	<---	PA	0.706	0.467	0.050	14.061	***	b		
Well-being	<---	Frailty	-0.674	-0.383	0.058	-11.535	***		-0.075	***
PA	<---	WSA*frailty	0.030	0.394	0.003	9.877	***	c		

***p < 0.001; SE – standard error (of B); B – unstandardised regression weight; β – standardised regression weight; PA – physical activity; WSA – workplace social activity

In Table 2, frailty has a direct negative association (β = -0.383; critical ratio = -11.535; *p* < 0.001) and an indirect negative association (through PA) (β = -0.075; *p* < 0.001) with well-being in the higher income sample. In the low-income sample, frailty has no direct association with well-being but has an indirect negative association with well-being (β = -0.067; *p* < 0.001). Thus, PA fully mediates the association of frailty with well-being in the low-income sample but partially mediates this relationship in the higher-income sample. In both samples, frailty has a negative relationship with PA, which means that higher frailty was associated with lower PA. WSA moderates the frailty-PA relationship in the sense that frailty has a positive association with PA at higher WSA. PA had a positive association with well-being in both samples, suggesting that higher PA was associated with higher well-being in the low- and high-income samples.

**Table 3:** Estimates of moderated mediation by workplace social activity in the low and higher-income samples

Parameter	Low income (*n* = 530)				Higher income (*n* = 897)			
		95% CI				95% CI		
	B	Lower	Upper	*p*	B	Lower	Upper	*p*
Simple slopes at three levels of WSA								
LowSS	-0.321	-0.380	-0.264	***	-0.369	-0.455	-0.290	***
MedSS	-0.128	-0.184	-0.072	***	-0.187	-0.276	-0.096	***
HighSS	0.065	-0.005	0.137	0.066	-0.004	-0.118	0.112	0.958
Conditional indirect effects at three levels of WSA								
LowCIE	-0.219	-0.272	-0.171	***	-0.261	-0.329	-0.196	***
MedCIE	-0.087	-0.130	-0.048	***	-0.132	-0.199	-0.069	***
HighCIE	0.045	-0.003	0.094	0.066	-0.003	-0.083	0.081	0.958
Index of moderated mediation								
InModMed	0.030	0.024	0.036	***	0.021	0.015	0.027	***

***p < 0.001; CI – confidence interval (of B, based on 2000 bias-corrected bootstrap iterations); B – unstandardized regression weight; SS – simple slope; CIE – conditional indirect effect; InModMed – index of moderated mediation; WSA – workplace social activity; please refer to Appendix 3b for formulae used to calculate the simple slopes and conditional indirect effects

**Fig. 4:**
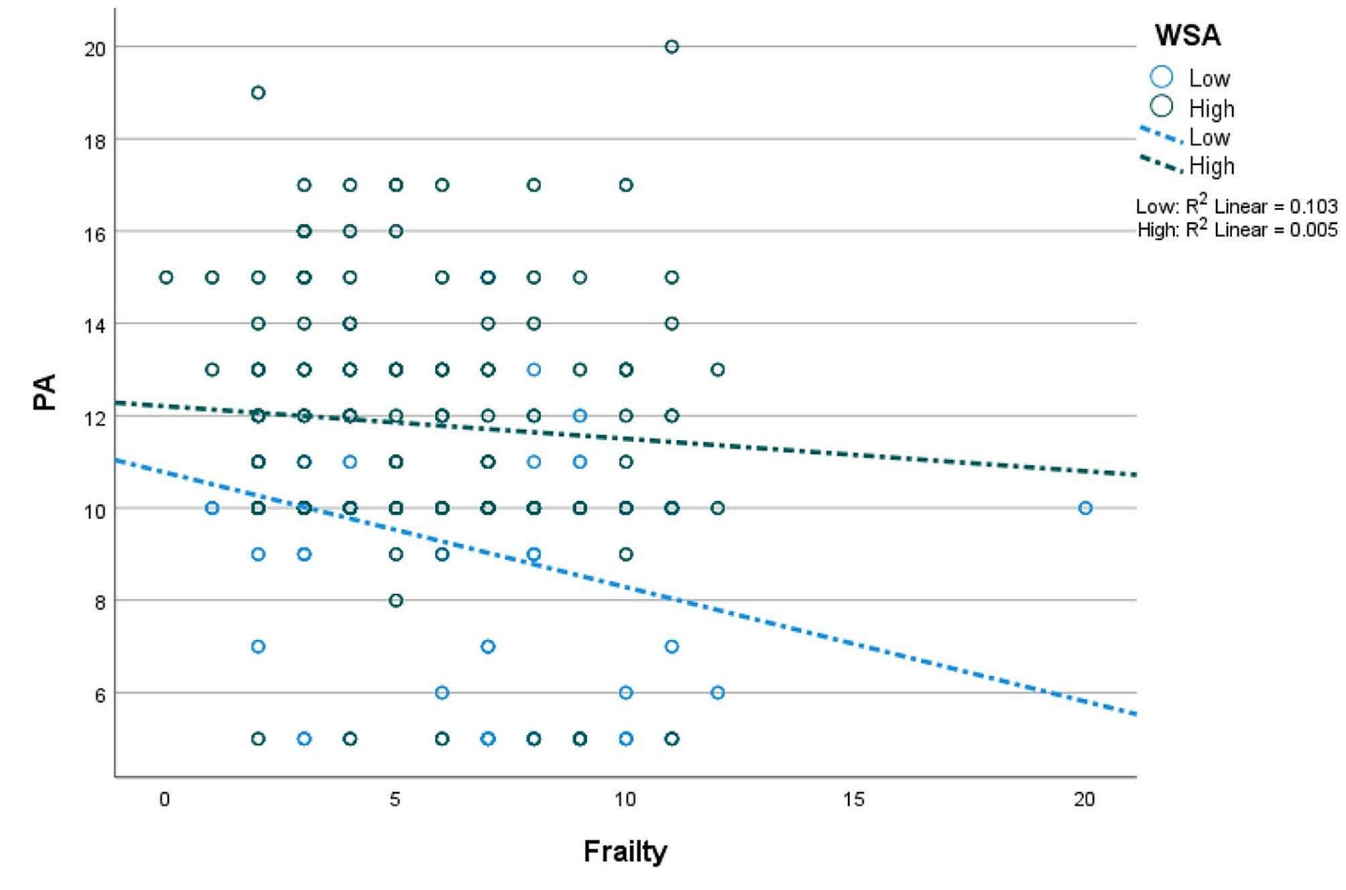
Association of frailty with physical activity at different levels (i.e., low = 448; high = 449) of WSA. *Note* WSA – workplace social activity; PA – physical activity

**Fig. 5:**
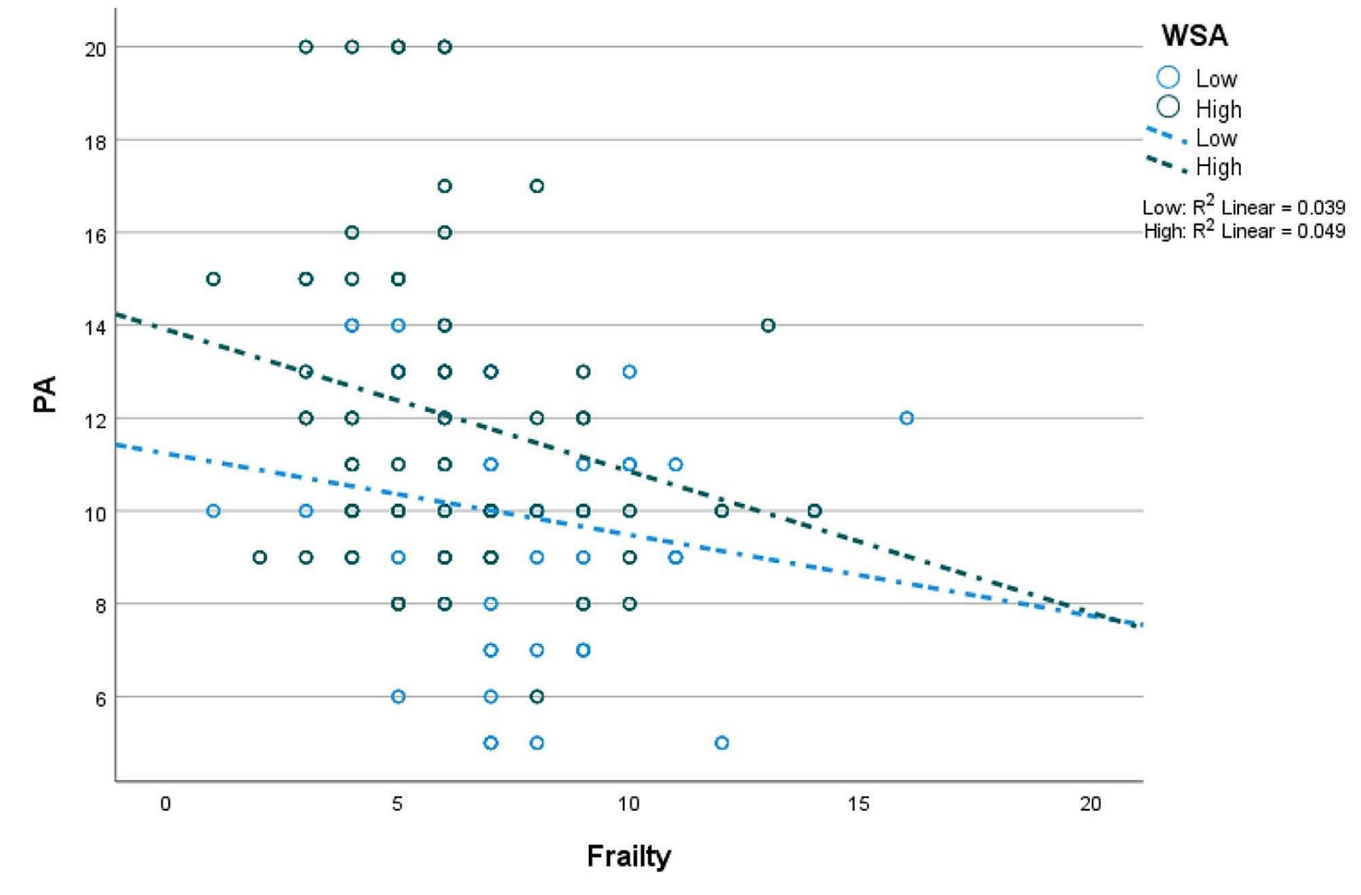
Association of frailty with physical activity at different levels (i.e., low = 265; high = 265) of WSA within the higher income sample. *Note* WSA – workplace social activity; PA – physical activity

In Table 3, there is evidence of moderated mediation in the low-income (unstandardised effect [B] = 0.030; *p* < 0.001) and higher-income (B = 0.027; *p* < 0.001) samples. This result suggests that frailty has a positive indirect association with well-being (through PA) at higher WSA in both samples. The moderating effect of WSA on the frailty-PA linkage is shown in Figs. 4 and 5. In Fig. 4, the negative association of frailty with PA is weaker at high WSA in the low-income sample. In the high-income sample, the negative association of frailty with PA is stronger at high WSA (see Fig. 5). Noteworthy is the evidence regarding the mediation of the frailty-well-being relationship by PA; this mediation is complete (i.e., full) in the low-income sample but partial in the higher-income sample.

## Discussion

This study aimed to examine a moderated mediation of PA in the nexus between frailty, WSA, and well-being. Two samples of older employees were compared in assessing this moderated mediation.

Frailty had a direct negative association with PA in only the higher income sample, which suggests that older employees with higher frailty reported lower PA behaviour. This result supports the idea that frailty with its physiological limitation can make it difficult for people to make the relevant energy expenditures in PA. For this reason, employees with higher frailty may be discouraged from performing PA regularly. This thought resonates with the basic import of the Disengagement Theory of Ageing (DTA), which asserts that people lose the physical strength or capability needed for PA as they age and, therefore, lose the ability to perform PA in later life [4]. The DTA, thus, suggests that the onset and progression of frailty are a consequence of ageing that would limit PA. Supporting this argument and our result are results from previous studies. A study in Canada, for example, has confirmed a negative association between frailty and PA despite using a different PA measurement method [5]. Other studies and systematic reviews [6, 7] utilising different measurement methods affirm our results.

Frailty was further found to have an indirect negative association with well-being through PA, although PA had a positive association with well-being in both samples. Thus, though PA can be associated with higher well-being, it rather transmits a negative influence from frailty to well-being. In other words, employees with higher frailty may experience poor well-being even if their PA directly improves their well-being. The above mediation of PA is partial [35, 36] in the higher-income sample, which means that frailty can predict well-being directly and does not need the mediation of PA to influence well-being. The partial mediation also signifies that there are two paths or channels along which frailty can influence well-being negatively. A full mediation in the low-income sample, on the other hand, implies that there is only one channel (the indirect path) in the model through which the independent variable (i.e., frailty) affects the dependent variable (i.e., well-being). Deductively, frailty can be a double-impact risk for poor well-being in only the higher income sample, whereby the primary impact is its direct negative association with well-being and the secondary impact is its indirect negative association with well-being. It is worth noting that other variables apart from PA can mediate the relationship between frailty and well-being, although a consideration of such variables was beyond the scope of this study.

The study found a moderated mediation of PA in the relationship between frailty, WSA, and well-being. This result has two parts, with the first part being the moderating role of WSA in the frailty-PA relationship. This moderation suggests that frailty less influences PA negatively at higher WSA in both samples. WSA such as interactions with peers or walking to a lounge in groups do not necessarily require physical strength or energy expenditure needed in vigorous PA. To explain, older employees with frailty who perform such activities are likely to maintain PA, especially moderate-intensity PA (e.g., walking). What makes our evidence compelling is that the association of frailty with PA can become positive at higher WSA in both samples. This moderating role of WSA is rooted in the buffering influence of WSA on frailty, which is implicit in the interaction influence (i.e., WSA*frailty). The association of social activity with frailty has been confirmed in several previous studies [22–24, 37], and noteworthy is the negative influence of social activity on frailty confirmed in a study conducted in Japan [22]. The current study provides unique evidence by being the first to confirm the frailty-social-activity relationship in a work context.

The second part of the moderated mediation is the significant moderated mediation index (see Table 3), which signifies that PA mediates the association of frailty with well-being at different levels of WSA. More specifically, the indirect negative association of frailty with well-being is smaller at higher WSA, which means that WSA does not only reduce the negative effect of frailty on PA but also reduces the indirect negative effect of frailty on well-being. This evidence is an extension of previous research confirming social engagement as a health behaviour that can buffer frailty or attenuate its negative effect on health outcomes [24, 37]. It constitutes an extension of the debate because previous research has only confirmed social activity as a direct predictor of PA and well-being, not as a moderator in the context of a moderated mediation. Moreover, this study is the first to consider social activity in a work context.

Worth noting as a unique element of this study are differences in the results between the two samples. For instance, a full mediation was confirmed in the low-income sample, but a partial mediation was confirmed in the higher-income sample. These differences suggest that the nature of the association of frailty with PA and well-being in the moderated mediation model may not be the same for different income groups. The existing evidence suggests that frailty can differ across income groups [3, 25], and this study builds upon this evidence by suggesting that frailty can be differently related to health behaviours (i.e., WSA and PA) and well-being in different income groups. This contribution of the study recalls the possibility of healthcare necessitated by poor well-being being differently predicted by frailty in different income groups. Since the negative association of frailty with well-being in the higher-income sample is stronger, it can be inferred that older employees in this income group, compared with those in the low-income group, would have a higher healthcare need.

The foregoing thought unfolds two practical implications. First, employers are more likely to spend on employees’ health insurance and record absences due to ill-health in the higher income group. This knowledge may enable organizations to plan for the healthcare of groups with different incomes. Since the burden of healthcare is higher among people with higher frailty [38, 39], health service providers may face a higher burden of care from the higher income sample, which is why knowledge about the income distribution of patients with frailty may facilitate the preparedness of healthcare providers. Since frailty more strongly predicts poorer PA and well-being in the higher income sample, our evidence implies an opportunity for organizations to support their employees to avoid frailty and its adverse outcomes (e.g., insufficient PA and poor well-being) through rewards schemes. To better understand this opportunity, future qualitative studies should investigate why frailty more strongly predicts lower PA and well-being in the higher-income group. These studies may provide an understanding of how employees may be rewarded financially in their frailty management programmes.

### Limitations and strengths

This study has some limitations that future researchers and decision-makers should consider. Due to research constraints beyond our control, we had to select participants with non-probability sampling and limit the samples to Accra. For this reason, the evidence from this study may have limited generalizability. Future studies utilising larger representative samples can fill this gap and advance our evidence. The cross-sectional design provides effect sizes representing only associations, which means it does not imply causation between the variables. Tools used to measure the study variables are subjective [as they are based on self-report] and vulnerable to response bias. Studies utilising objective measures where necessary are, therefore, needed. Future research may enhance our evidence by using objective PA measures (e.g., activity trackers such as pedometers and accelerometers).

As mentioned earlier, several other variables not considered in this study may mediate the association of frailty with well-being. Since these potential mediating roles may provide implications for practice, future researchers are encouraged to assess them. Similarly, future research should consider other possible moderators of the association between frailty and PA. WSA is major part of employees’ daily routine and can, therefore, be expected to influence PA and well-being within organisations. Yet, this study was among a few studies that have assessed the association of WSA with frailty, PA, and well-being. Given this shortcoming of the literature, more future research on WSA and how it relates with health-related variables is needed.

Against the above limitations are several strengths of this study. This study was the first to assess a moderated mediation of PA in the relationship between frailty, WSA, and well-being in a work context. Previous research has focused on social activity outside the workplace, which means that this study provides evidence that may be considered in the design and implementation of workplace health promotion and ageing policies. The cross-sectional design utilised meets items on the STROBE checklist (see Appendix 4), which means this study met relevant quality criteria. We adopted a sensitivity analysis to screen for the ultimate covariates based on previous research [27, 32], which enabled us to avoid adjusting for irrelevant variables assumed to be confounders. Finally, our design was strengthened by our comparison of two samples of older employees with different income levels. This analysis provided insights into how the moderated mediation by WSA may vary between groups with different income levels.

## Conclusions

In the low-income sample, frailty had only an indirect negative association with well-being, which signifies a full mediation of PA in the frailty-well-being relationship. In the higher income sample, frailty had a negative direct association and a negative indirect association through PA on well-being, which signifies a partial mediation of PA. Though frailty had a negative association with PA in both samples, it had a positive association with PA at higher WSA. It is, therefore, concluded that WSA moderated the effect of frailty on PA in the sense that it was associated with higher PA at higher WSA. There was evidence of moderated mediation, which means that the indirect negative effect of frailty on well-being was weaker at higher WSA. It is concluded that WSA may lower the likelihood of frailty predicting lower PA and well-being. Workplace interventions aimed at enhancing WSA may encourage PA and improve well-being among older employees with frailty.

### Electronic supplementary material

Below is the link to the electronic supplementary material.

Supplementary Material 1

Supplementary Material 2

Supplementary Material 3

Supplementary Material 4

Supplementary Material 5

Supplementary Material 6

## Data Availability

No datasets were generated or analysed during the current study.
