# Supplementary material for: Association of frailty with workplace social activity, physical activity, and well-being among older employees: a moderated mediation in two income-variant samples
Source: BMC Geriatr. 2024 Jul 3;24:574. doi: 10.1186/s12877-024-05178-9 (PMC11223269; doi:10.1186/s12877-024-05178-9)
Supplement: Supplementary file 3 — Supplementary Material 3 [file 12877_2024_5178_MOESM3_ESM.doc]

Appendix 1. Scales and items used to measure the main variables

**Appendix 1a. Items for measuring frailty**

| No. | Question | Response | |
| --- | --- | --- | --- |
|  |  | No (0) | Yes (1) |
| 1 | Do you feel physically healthy? |  |  |
| 2 | Have you lost a lot of weight recently without wishing to do so? |  |  |
| 3 | Do you experience problems in your daily life due to difficulty in walking? |  |  |
| 4 | Do you experience problems in your daily life due to difficulty maintaining your balance? |  |  |
| 5 | Do you experience problems in your daily life due to poor hearing? |  |  |
| 6 | Do you experience problems in your daily life due to poor vision? |  |  |
| 7 | Do you experience problems in your daily life due to a lack of strength in your hands? |  |  |
| 8 | Do you experience problems in your daily life due to physical tiredness? |  |  |
| 9 | Do you have problems with your memory? |  |  |
| 10 | Have you felt down during the last month? |  |  |
| 11 | Have you felt nervous or anxious during the last month? |  |  |
| 12 | Are you able to cope with problems well? |  |  |
| 13 | Do you live alone? |  |  |
| 14 | Do you sometimes miss having people around you? |  |  |
| 15 | Do you receive enough support from other people? |  |  |

**Appendix 1b. Items for measuring physical activity**

| No | Statement | 1 | 2 | 3 | 4 |
| --- | --- | --- | --- | --- | --- |
| 1 | Exercised vigorously for 20 or more minutes at least three times a week (such as brisk walking, bicycling, aerobic dancing, and using a stair climber) |  |  |  |  |
| 2 | Took part in light to moderate physical activity (such as walking 30-40 minutes 5 or more times a week. |  |  |  |  |
| 3 | Took part in leisure-time (recreational) physical activities (such as dancing, swimming, and bicycling) |  |  |  |  |
| 4 | Did stretching exercises at least 3 times per week. |  |  |  |  |
| 5 | Got exercise during usual daily activities (such as walking during lunch, using stairs instead of elevators, and parking car away from destination and walking). |  |  |  |  |

Note: Descriptive anchors are 1 – never, 2 – sometimes, 3 – often, and 4 – routinely

**Appendix 1c. Items for measuring well-being**

| # | Item | 1 | 2 | 3 | 4 | 5 |
| --- | --- | --- | --- | --- | --- | --- |
| 1 | I have felt cheerful and in good spirits |  |  |  |  |  |
| 2 | I have felt calm and relaxed |  |  |  |  |  |
| 3 | I have felt active and vigorous |  |  |  |  |  |
| 4 | I woke up feeling fresh and rested |  |  |  |  |  |
| 5 | My daily life has been filled with things that interest me |  |  |  |  |  |

Note: descriptive anchors are never – 1; sometimes – 2; often – 3, very often – 4, and all the time – 5.

**Appendix 1d. Items for measuring workplace social activity**

| No. | Item | 1 | 2 | 3 | 4 |
| --- | --- | --- | --- | --- | --- |
| 1 | I interacted with colleagues or friends at work |  |  |  |  |
| 2 | I played a game or performed a sporting activity (e.g., walking) with colleagues or friends at work |  |  |  |  |
| 3 | I went to a performance-based event (e.g., seminar, conference, training) organized at work |  |  |  |  |
| 4 | Provide help to friends, colleagues or workmates at work |  |  |  |  |
| 5 | I catered for a colleague or friend who was disadvantaged (e.g., disabled or had a special need) at work |  |  |  |  |
| 6 | I made new friends at work |  |  |  |  |
| 7 | I participated in non-work-related or recreational events with a friend or colleague at work |  |  |  |  |
| 8 | I participated in voluntary or unpaid activities at work with others to contribute to the overall success of my organization |  |  |  |  |

Note: The descriptive anchors are 1 – not at all, 2 – less frequently, 3 – frequently, and 4 – very frequently
