# Supplementary material for: Association of frailty with workplace social activity, physical activity, and well-being among older employees: a moderated mediation in two income-variant samples
Source: BMC Geriatr. 2024 Jul 3;24:574. doi: 10.1186/s12877-024-05178-9 (PMC11223269; doi:10.1186/s12877-024-05178-9)
Supplement: Supplementary file 4 — Supplementary Material 4 [file 12877_2024_5178_MOESM4_ESM.doc]

**Appendix 2. Factors extracted and variance explained on each scale (consolidated data)**

| **Appendix 2a. Total Variance Explained (Frailty)** | | | | | | | | | |
| --- | --- | --- | --- | --- | --- | --- | --- | --- | --- |
| Component | Initial Eigenvalues | | | Extraction Sums of Squared Loadings | | | Rotation Sums of Squared Loadings | | |
|  | Total | % of Variance | Cumulative % | Total | % of Variance | Cumulative % | Total | % of Variance | Cumulative % |
| 1 | 3.842 | 25.614 | 25.614 | 3.842 | 25.614 | 25.614 | 2.448 | 16.320 | 16.320 |
| 2 | 1.381 | 9.207 | 34.821 | 1.381 | 9.207 | 34.821 | 2.440 | 16.267 | 32.588 |
| 3 | 1.307 | 8.713 | 43.534 | 1.307 | 8.713 | 43.534 | 1.464 | 9.761 | 42.348 |
| 4 | 1.130 | 7.531 | 51.065 | 1.130 | 7.531 | 51.065 | 1.197 | 7.982 | 50.331 |
| 5 | 1.070 | 7.130 | 58.195 | 1.070 | 7.130 | 58.195 | 1.180 | 7.864 | 58.195 |
| 6 | .935 | 6.232 | 64.427 |  |  |  |  |  |  |
| 7 | .882 | 5.879 | 70.306 |  |  |  |  |  |  |
| 8 | .822 | 5.481 | 75.786 |  |  |  |  |  |  |
| 9 | .710 | 4.737 | 80.523 |  |  |  |  |  |  |
| 10 | .606 | 4.037 | 84.560 |  |  |  |  |  |  |
| 11 | .551 | 3.675 | 88.235 |  |  |  |  |  |  |
| 12 | .506 | 3.370 | 91.605 |  |  |  |  |  |  |
| 13 | .460 | 3.069 | 94.674 |  |  |  |  |  |  |
| 14 | .459 | 3.058 | 97.732 |  |  |  |  |  |  |
| 15 | .340 | 2.268 | 100.000 |  |  |  |  |  |  |
| Extraction Method: Principal Component Analysis. | | | | | | | | | |

| **Appendix 2b. Total Variance Explained (Physical activity)** | | | | | | |
| --- | --- | --- | --- | --- | --- | --- |
| Component | Initial Eigenvalues | | | Extraction Sums of Squared Loadings | | |
|  | Total | % of Variance | Cumulative % | Total | % of Variance | Cumulative % |
| 1 | 2.844 | 36.889 | 36.889 | 2.844 | 36.889 | 36.889 |
| 2 | .989 | 29.772 | 66.661 |  |  |  |
| 3 | .534 | 20.684 | 87.345 |  |  |  |
| 4 | .417 | 8.331 | 95.676 |  |  |  |
| 5 | .216 | 4.324 | 100.000 |  |  |  |
| Extraction Method: Principal Component Analysis. | | | | | | |

| **Appendix 2c. Total Variance Explained (Well-being)** | | | | | | |
| --- | --- | --- | --- | --- | --- | --- |
| Component | Initial Eigenvalues | | | Extraction Sums of Squared Loadings | | |
|  | Total | % of Variance | Cumulative % | Total | % of Variance | Cumulative % |
| 1 | 4.178 | 43.570 | 43.570 | 4.178 | 43.570 | 43.570 |
| 2 | 0.295 | 25.909 | 69.479 |  |  |  |
| 3 | 0.215 | 24.298 | 93.777 |  |  |  |
| 4 | 0.196 | 3.912 | 97.689 |  |  |  |
| 5 | 0.116 | 2.311 | 100.000 |  |  |  |
| Extraction Method: Principal Component Analysis. | | | | | | |

| **Appendix 2d. Total Variance Explained (Workplace Social Activity)** | | | | | | |
| --- | --- | --- | --- | --- | --- | --- |
| Component | Initial Eigenvalues | | | Extraction Sums of Squared Loadings | | |
|  | Total | % of Variance | Cumulative % | Total | % of Variance | Cumulative % |
| 1 | 4.962 | 32.023 | 62.023 | 4.962 | 32.023 | 32.023 |
| 2 | .948 | 21.850 | 73.873 |  |  |  |
| 3 | .627 | 17.840 | 81.713 |  |  |  |
| 4 | .496 | 16.204 | 87.917 |  |  |  |
| 5 | .341 | 4.260 | 92.177 |  |  |  |
| 6 | .285 | 3.564 | 95.741 |  |  |  |
| 7 | .196 | 2.452 | 98.193 |  |  |  |
| 8 | .145 | 1.807 | 100.000 |  |  |  |
| Extraction Method: Principal Component Analysis. | | | | | | |
