# Supplementary material for: Association of frailty with workplace social activity, physical activity, and well-being among older employees: a moderated mediation in two income-variant samples
Source: BMC Geriatr. 2024 Jul 3;24:574. doi: 10.1186/s12877-024-05178-9 (PMC11223269; doi:10.1186/s12877-024-05178-9)
Supplement: Supplementary file 5 — Supplementary Material 5 [file 12877_2024_5178_MOESM5_ESM.doc]

**Appendix 3**

**Appendix 3a. Steps taken in the sensitivity analysis for confounding variables**

| Stage | # | Action (performed on each of the two samples) |
| --- | --- | --- |
| 1 | 1 | Fit a simple linear regression model to assess the relationship between frailty and wellbeing |
|  | 2 | Note the standardised regression weight from step 1 |
|  | 3 | Fit a multiple linear regression model in which all measured confounding variables are treated as predictors of frailty, the main predictor |
|  | 4 | Identify from step 3 potential confounders that have a p-value ≥0.25 |
|  | 5 | Predictors from step 4 that produced a p≥0.25 should be removed from the analysis and the others kept for the next stage of the analysis |
| 2 | 6 | Adjust for each of the remaining confounding variables in the model fitted at step 1 |
|  | 7 | Compute the per cent (%) change between the standardised regression weight at step 1 and the new weight resulting from step 6 |
|  | 8 | All potential confounders that produce a change of 10% or more should be incorporated into the final analysis as the ultimate confounders |

**Appendix 3b. Equations used to estimate the index of moderated mediation**

Key statistics from the data:

1. mean of centred moderator (i.e., workplace social activity (WSA)) for low-income sample = 0
2. standard deviation (SD) of the moderator for the low-income sample = 4.34764
3. mean of moderator (centred) for higher-income sample = 0
4. standard deviation of the moderator for the higher-income sample = 6.01313

**General equations**

lowSS=a+c*(mean – 1SD)

medSS=a+b*(mean)

highSS=a+c*(mean + 1SD)

**Equations based on data from the low-income sample**

lowSS=a+c*(-4.34764)

medSS=a

highSS=a+c*(4.34764)

**Equations based on data from the higher-income sample**

lowSS=a+c*(-6.01313)

medSS=a

highSS=a+c*(6.01313)

**Stage 2: estimation of the conditional indirect effects based on estimated simple slopes**

lowCIE=lowSS*b

medCIE=medSS*b

highCIE=highSS*b

**Stage 3: estimation of the index of moderated mediation**

InModMed=c*b

**Note**: please see the statistical model tested to make sense of these equations. * represents multiplication
